# Health-related Quality of Life and Mental Health of Elderly by Occupational Status

**Published:** 2017-08

**Authors:** Yeunhee KWAK, Yoonjung KIM

**Affiliations:** Red Cross College of Nursing, Chung-Ang University, Seoul, Republic of Korea

**Keywords:** Quality of life, Mental health, Employment, Aged

## Abstract

**Background:**

This study aimed to identify the association between health-related quality of life and mental health by elderly Koreans’ occupational status.

**Methods:**

This descriptive cross-sectional study consisted of a secondary analysis of data from the Korea National Health and Nutrition Examination Survey V-3 (2012). The sample comprised 1431 people aged 65 yr and older.

**Results:**

Compared to participants employed, those not showed lower HRQOL. Occupational status significantly affected all of the EuroQoL-5 Dimensions (EQ-5D): mobility, self-care, usual activity, pain/discomfort, and anxiety/depression. Unemployed participants had more suicidal ideation. Among employed elderly persons, significant differences were found between manual and non-manual workers in the EQ-5D index and EQ-5D for mobility and pain/discomfort. Manual workers experienced more depression and suicidal ideation than did non-manual workers.

**Conclusion:**

The occupational status of elderly individuals accounts for differences in their quality of life and mental health status. Therefore, additional jobs should be created for the elderly in order to improve their quality of life and mental health.

## Introduction

According to population estimates from the Korea National Statistical Office (KNSO), South Korea is currently an aging society, in which the elderly population aged 65 yr and older accounts for 13.1% of the total population. It is expected to become an aged society by 2019, as the elderly will reach 14% of the total population (1). South Korea is aging more rapidly than are many other countries in the world. As the population to support the elderly is decreasing because of reduced birth rates, burdens on families and society as a whole are increasing. The cost of supporting the elderly population has become a societal issue. As a result, the elderly are facing social pressure to continue participating in economic activities and to secure financial independence. According to one study, financial independence has a significant correlation with mental health and quality of life (QOL) (2, 3). In fact, social activities of the elderly include volunteering and religious activities, but economic activities have the strongest influence on their lives (4).

Productive activities during old age play an important role in society. Many studies have examined the effect of employment and other productive activities on mental health among the elderly, including depression and well-being. These empirical studies, mostly conducted in the West, report that elderly subjects’ participation in productive activities has a positive effect on them, such as reducing depression and stress (5–10). In addition, job loss and subsequent financial difficulty can lead to suicidal ideation. Therefore, the retired, unemployed elderly are more likely to commit suicide than are those employed (9, 11, 12). However, few studies have specifically addressed the correlations between the occupational status of the elderly and their mental health.

Health-related QOL (HRQOL) (13) refers to perceived QOL that is directly related to an individual’s health. It is a subjective and multidimensional concept as well as a useful index representing the health of individuals or populations that can be used to assess daily function and well-being among the elderly (3). Factors related to HRQOL include economic status, marital status, educational level, activities of daily living, subjective physical symptoms, and health condition (3, 14). For the elderly, an occupation is an instrument for their social life, rather than for raising a family; economic status is considered an important factor that influences QOL among the elderly (15). For the elderly, employment improves QOL by increasing their confidence from taking part in the economy and improving their self-esteem, income, and general happiness as a result (6, 15). Ultimately, extending economic activities into old age is a critical element for improving QOL in the elderly. However, there has been little research concerning correlations between specific occupational status and elderly QOL. In addition, although HRQOL is included as a subcomponent of various aspects of QOL, it has rarely been analyzed.

This study was conducted in order to collect basic data to develop nursing interventions to improve elderly HRQOL and mental health. We analyzed the occupational status, HRQOL, and mental health of elderly adults aged 65 yr and older based on raw data from the Korea National Health and Nutrition Examination Survey (KNHANES) V-3, which is both representative and reliable.

## Materials and Methods

### Study design

This study employed a cross-sectional design to identify differences in HRQOL and mental health according to the occupational status in the South Korean elderly aged 蠅65 yr.

### KNHANES and the study population

The KNHANES has been performed since 1998 by the Korea Centers for Disease Control and Prevention (KCDC) to identify the health and nutrition status of Koreans. The KNHANES was performed after approval by the KCDC Institutional Review Board (IRB No. 2012-01EXP-01-2C). This cross-sectional study used raw data from the KNHANES V-3 under official approval of the relevant organization. The KNHANES is a nationally representative, cross-sectional survey targeting non-institutionalized Korean people. It consists of health, nutrition, and examination surveys. Health and nutrition surveys were conducted based on one-on-one interviews and self-report. The KCDC examination team performed the examination. Samples were collected by using a stratified, multi-staged, and clustered probability design in order to represent the entire South Korean population. In addition, the KNHANES V adopted a rolling survey sampling method so that each rolling sample of the relevant year becomes a probability sample that represents the entire country, and rolling samples have independent and homogeneous characteristics. In the year V-3 (2012), of 10,589 subjects, 8057 participated (participation rate: 80.0%) (16). Data from people aged 65 yr and older (n = 1666) from the KNHANES V-3 dataset (n = 8057) were selected. Of the 1666 subjects, data from 1431, excluding those with missing information in the questionnaire or examination, were used for the final analysis.

### Variables

#### HRQOL

For HRQOL, the EuroQoL-5 Dimensions (EQ-5D), EQ-5D index, and EuroQoL-visual analog scale (EQ-VAS) developed by the EuroQoL group were used (17). The EQ-5D is an instrument for measuring HRQOL designed to measure simple and overall health for clinical and economic efficiency evaluation. It assesses five dimensions (mobility, self-care, usual activities, pain/discomfort, and anxiety/depression). Each dimension can be answered on three levels: ‘no problems’, ‘some problems’, or ‘severe problems’. The EQ-5D index is calculated by weighing questions for the five dimensions; the scores are distributed between 1, meaning perfect health, and −1, meaning a poor health state that is considered worse than death (18). For the EQ-VAS, subjects were asked to mark their health status on a vertical line with gradation ranging from 0 (the worst imaginable state of health) to 100 (the best imaginable state of health).

### Mental health

To assess participants’ mental health, variables such as stress, depression, and suicide ideation were used. For stress, the participants answered the question, ‘How much stress do you feel in daily living?’ The answers ’very much ’ and ’much’ were categorized as ’yes’ and ’little’ and ’almost none’ as ’no’. For depression, answers to the question, ’During the past year, have you felt sad or frustrated to an extent that it interfered with your daily living for two weeks or longer?’ were categorized as ‘yes’ or ‘no’. To assess suicidal ideation, the participants answered to the question, ’Did you ever think that you wanted to die during the past year?’ as ’yes’ or ’no’.

### Occupational status

The occupational status of the participants was investigated based on employment status and type. For employment status, the participants answered the question, ‘During the past week, have you worked for one hour or more for the purpose of income, or worked for a family business for 18 h or longer without pay?’, as ‘yes’ or ‘no’. Working type was categorized by the major categories of the Korean Standard Classification of Occupations (19), based on the degree of physical work. Non-manual workers included managers, professionals and related workers, and clerks. Manual workers included service and sales workers, agricultural and fishery workers, craftspeople, mechanics, assembly workers, simple laborers, and soldiers.

### Covariates

Demographic variables of the participants included age, sex, body mass index (BMI), education, marital status, economic status, place of residence, smoking status, and drinking status. BMI was calculated based on the equation, weight (kg)/height (m)^2^. Education was categorized as elementary school or less, middle school, high school, and college or more and marital status as unmarried and married. Economic status was divided by quartile based on equivalent income, i.e., family income corrected by the number of family members (average monthly household income/√ (no. of household members)). Living place was categorized as urban or rural. For smoking status, participants who smoked 100 or more cigarettes throughout their life and currently smoked were categorized as ’yes’ and otherwise, ’no’. For drinking status, subjects who, during the past year, drank alcoholic beverages once or more per month were categorized as ’yes’ and otherwise, ’no’.

### Statistical analysis

All data were presented as mean ± SE for continuous variables or as n (percentage) for categorical variables. The SAS survey procedure (ver. 9.3; SAS Institute Inc., Cary NC, USA) was used to run a complex sample design based on data analysis from the survey data; this provided sampling weights of KNHANES and nationally representative estimates. The significance threshold was 0.05. Differences in occupational status by demographic characteristics were verified by using t-tests and χ^2^ tests. Differences in HRQOL and mental health by occupational status were also verified using t-tests and χ^2^tests. We performed a multiple regression analysis to identify factors related to elderly HRQOL and mental health. Relationships between HRQOL and demographic characteristics and occupational status were examined via multiple linear regression analyses; the relationships between mental health and the above factors were investigated by multiple logistic regression analyses.

## Results

### Occupational status by demographic characteristics

Differences in occupational status by demographic characteristics of the subjects are shown in Table 1. Of people aged 65 yr and older, 34% were working. The participants, who worked, were generally younger men with a low BMI. The employed subjects were significantly more likely than were unemployed subjects to live in rural areas, have a low level of education, and to smoke.

**Table 1:** Occupational status by demographic characteristics (mean ± SE or n (%))

**Characteristic**	**Employed (N = 1,431)**			**Employment type (N = 485)**						
**Group**	**Yes (N = 486)**	**No (N = 945)**	***P*-value**	**Non-manual (N = 37)**	**Manual (N = 448)**	***P*-value**
Age	71 ± 4.6	73.3 ± 5.4	< 0.001	70.0 ± 4.3	71.1 ± 4.6	0.153
Sex			< 0.001			0.184
Men	260 (53.5)	343 (36.3)		30 (81.0)	229 (51.1)	
Women	226 (46.5)	602 (63.7)		7 (19.0)	219 (48.9)	
BMI (kg/m^2^)	23.7 ± 2.9	24.1 ± 3.3	0.037	23.7 ± 2.8	23.7 ± 3.0	0.930
Residence			< 0.001			< 0.001
Urban	274 (56.4)	700 (74.1)		33 (89.2)	240 (53.6)	
Rural	212 (43.6)	245 (25.9)		4 (10.8)	208 (46.4)	
Education			0.038			< 0.001
Elementary	319 (65.6)	584 (61.8)		4 (10.8)	315 (70.3)	
Middle	72 (14.8)	116 (12.3)		5 (13.5)	67 (15.0)	
High	58 (11.9)	161 (17.0)		10 (27.0)	47 (10.5)	
University	37 (7.6)	84 (8.9)		18 (48.6)	19 (4.2)	
Marital status			0.690			0.500
Married	485 (99.8)	943 (99.9)		37 (100)	447 (99.8)	
Unmarried	1 (0.2)	1 (0.1)		0 (0)	1 (0.2)	
Economic status			0.067			< 0.001
Lowest	213 (44.4)	470 (50.3)		5 (13.5)	208 (47.1)	
Lower	156 (32.5)	243 (26)		14 (37.9)	142 (32.1)	
Intermediate higher	68 (14.2)	132 (14.1)		6 (16.2)	61 (13.8)	
Intermediate highest	43 (8.9)	89 (9.6)		12 (32.4)	31 (7.0)	
Smoking (yes)	77 (15.9)	82 (8.7)	< 0.001	4 (10.8)	72 (16.1)	0.400
Drinking (yes)	397 (81.7)	751 (79.5)	0.388	35 (94.6)	361 (80.6)	< 0.001

BMI, body mass index; SE, standard error.

The vast majority (92.4%) of workers were manual workers. Manual workers were more likely than non-manual workers to live in rural regions, have a low level of education, belong to the lowest economic class, and to be non-drinkers.

### Differences in health-related QOL and mental health by occupational status

Participants’ HRQOL and mental health by occupational status are presented in Table 2. Unemployed subjects had a lower HRQOL and mental health than did employed participants. The mean EQ-5D index of unemployed and employed participants differed significantly. All of the EQ-5D subsections (mobility, self-care, usual activity, pain/discomfort, and anxiety/depression) showed significant differences by occupational status. The EQ-VAS score did not show a statistically significant difference. Unemployed subjects were more likely than those employed to have suicidal ideation were. No statistically significant differences were found for stress and depression.

**Table 2:** Health-related quality of life and mental health by occupational status (mean ± SE or n (%))

**Characteristic**	**Employed (N = 1,431)**			**Employment type (N = 485)**						
Group	**Yes (N = 486)**	**No (N = 945)**	***P*** **-value**	**Non-manual (N = 37)**	**Manual (N = 448)**	***P*-value**
EQ-5D Mobility			0.011			0.045
No problem	318 (65.4)	541 (57.2)		31 (83.8)	286 (63.8)	
Some problem	158 (32.5)	377 (39.9)		6 (16.2)	152 (33.9)	
Severe problem	10 (2.1)	27 (2.9)		0 (0.0)	10 (2.2)	
Self-care			0.003			0.142
No problem	443 (91.2)	796 (84.2)		37 (100.0)	405 (90.4)	
Some problem	40 (8.2)	134 (14.2)		0 (0.0)	40 (8.9)	
Severe problem	3 (0.6)	14 (1.5)		0 (0.0)	3 (0.7)	
Usual activity			<0.001			0.066
No problem	407 (83.7)	680 (72.0)		36 (97.3)	370 (82.6)	
Some problem	73 (15.0)	226 (23.9)		1 (2.7)	72 (16.1)	
Severe problem	6 (1.2)	37 (3.9)		0 (0.0)	6 (1.3)	
Pain/discomfort			<0.001			0.008
No problem	337 (69.3)	546 (57.8)		34 (91.9)	302 (67.4)	
Some problem	127 (26.1)	312 (33)		3 (8.1)	124 (27.7)	
Severe problem	22 (4.5)	87 (9.2)		0 (0.0)	22 (4.9)	
Anxiety/depression			0.004			0.104
No problem	415 (85.4)	740 (78.3)		35 (94.6)	379 (84.6)	
Some problem	65 (13.4)	182 (19.3)		1 (2.7)	64 (14.3)	
Severe problem	6 (1.2)	23 (2.4)		1 (2.7)	5 (1.1)	
EQ-5D index	0.9 ± 0.2	0.8 ± 0.2	<0.001	1 ± 0.1	0.9 ± 0.2	<0.001
EQ-VAS	76.5 ± 87.1	76.6 ± 107.2	0.991	80.7 ± 11.6	76.2 ± 90.7	0.336
Stress			0.428			0.066
No	389 (80.4)	737 (78.6)		34 (91.9)	354 (79.4)	
Yes	95 (19.6)	201 (21.4)		3 (8.1)	92 (20.6)	
Depression			0.104			0.049
No	421 (86.8)	785 (83.5)		36 (97.3)	384 (85.9)	
Yes	64 (13.2)	155 (16.5)		1 (2.7)	63 (14.1)	
Suicidal idea			0.002			0.028
No	411 (84.9)	735 (78.1)		36 (97.3)	374 (83.9)	
Yes	73 (15.1)	206 (21.9)		1 (2.7)	72 (16.1)	

EQ-5D, EuroQoL 5 dimensions; EQ-VAS, EuroQoL visual analog scale; SE, standard error

As for employed elderly adults’ HRQOL and mental health by working type, manual workers showed lower HRQOL and mental health than did non-manual workers. The mean EQ-5D index of manual workers was significantly higher than that of non-manual workers. Of the EQ-5D subsections, mobility and pain/discomfort were different between manual workers and non-manual workers. In addition, in terms of mental health, more manual workers than non-manual workers experienced depression and suicidal ideation. No statistically significant difference was found for EQ-VAS and stress.

### Factors associated with health-related QoL according to occupational status

Table 3 lists factors related to HRQOL of people aged 65 yr and older. Of HRQOL subsections, only the EQ-5D index was found to be statistically significant. Among people aged 65 yr and older, age, male sex, working, smoking, and drinking were negatively correlated with HRQOL. Educational levels were positively correlated with HRQOL.

**Table 3:** Factors Associated Health-related QOL According to Occupational Status^a^

	**EQ-5D index**						
	**Elderly**			**Working elderly**						
	**β**	**SE**	***P***	**β**	**SE**	***P***
Intercept	1.303	0.073	< .001	1.265	0.138	< .001
Age	−0.003	0.001	< .001	−0.002	0.002	0.074
Men (vs. women)	−0.057	0.012	< .001	−0.055	0.018	0.003
Working (vs. yes)	−0.038	0.010	< .001			
Working type (vs. Non-manual)				−0.023	0.030	0.433
Edu1	0.046	0.014	0.001	0.015	0.021	0.466
Edu2	0.070	0.014	< .001	0.057	0.024	0.018
Edu3	0.096	0.018	< .001	0.057	0.032	0.075
Smoking (vs. no)	−0.044	0.015	0.004	−0.021	0.021	0.311
Drinking (vs. no)	−0.033	0.013	0.01	−0.031	0.021	0.142

a Calculated by multiple linear regression, with data adjusted by all variables in the table.

Edu1 : middle school (vs. Elementary school) / Edu2 : high school (vs. Middle school) / Edu3 : university (vs. High school)

In the analysis that included only employed subjects, HRQOL was negatively correlated with male sex and positively correlated with high school education level.

Among the employed elderly, age, working type, other education levels, smoking, and drinking did not show significant differences.

### Factors associated with mental health according to occupational status

Table 4 lists factors related to mental health of people aged 65 yr and older. Of the mental health subsections, only depression and suicidal ideation were found to be statistically significant.

**Table 4:** Factors associated with mental health according to occupational status^a^ (Adjusted OR (95% CI))

	**Elderly**		**Working elderly**	
	**Depression**	**Suicidal ideation**	**Depression**	**Suicidal ideation**
Age	1.007(0.978–1.037)	1.002(0.976–1.029)	1.026(0.967–1.087)	0.943(0.888–1.002)
Men (vs. women)	2.186(1.455–3.284)	2.056 (1.414–2.991)	1.714(0.838–3.508)	1.672(0.825–3.385)
Working (vs. yes)	1.214(0.869–1.697)	1.615(1.179–2.213)		
Working type (vs. Non-manual)			3.429(0.429–27.427)	4.269(0.538–33.892)
Edu1	0.693(0.419–1.148)	0.517(0.321–0.831)	1.330(0.622–2.842)	0.818(0.378–1.769)
Edu2	0.918(0.584–1.442)	0.502(0.318–0.793)	0.822 (0.293–2.306)	0.829(0.333–2.063)
Edu3	0.399(0.178–0.896)	0.325(0.157–0.669)	0.344(0.042–2.812)	0.275(0.034–2.215)
Smoking (vs. no)	2.323(1.433–3.766)	3.078(1.975–4.798)	1.932(0.888–4.201)	2.639(1.273–5.469)
Drinking (vs. no)	1.019 (0.703–1.478)	0.990(0.702–1.395)	1.012 (0.485–2.112)	0.917(0.455–1.847)

a Calculated by multiple logistic regression, with data adjusted by all variables in the table.

CI: confidence interval; Edu1: middle school (vs. elementary school); Edu2: high school (vs. middle school); Edu3: university (vs. high school); or: odds ratio.

Among people aged 65 yr and older, men had a higher risk of depression and suicidal ideation than did women. Unemployed participants had a higher risk of suicidal ideation than did employed subjects. Middle and high school education levels were related to a lower risk of suicidal ideation. University education level was correlated with lower risk of depression and suicidal ideation. Smoking was related to a higher risk of depression and suicidal ideation than was non-smoking. In the analysis that included only employed subjects, only smoking was related to a higher risk of suicidal ideation than non-smoking. Other factors were not significantly associated with depression and suicidal ideation.

## Discussion

The association of HRQOL and mental health by occupational status observed in this study is expected to provide basic data for improving elderly HRQOL and mental health. This study showed that only 34% of people aged 65 yr and older were employed, and 92.4% of the employed were manual workers who engaged in physical labor. According to statistical data, the employment rate of the elderly in South Korea decreased from 34.0% in 2011 to 28.9% in 2014. However, the figure is still higher than the average elderly employment rate (12.3%) among countries in the Organization for Economic Co-operation and Development (1). In addition, 52.9% of the elderly in 2011 and 36.4% in 2014 were working in the agricultural and fishery industries; other simple and physical laborers showed tendencies similar to this study’s findings (1). Although the percentage of economic participation by the South Korean elderly is increasing, a high percentage of them are self-employed or, with respect to the working type and employment type, simple laborers, and temporary employees. This suggests a very low level of employment quality (20). The increasing elderly employment rate involves a positive aspect, i.e., expanded elderly participation in productive activities, and negative aspects, such as lack of preparation for income during old age and inadequate public systems for elderly financial support.

In this study, the unemployed elderly showed lower HRQOL and mental health than did employ ones. In particular, all of the EQ-5D, the EQ-5D index, and suicidal ideation showed significant differences. Elderly employment was improved QOL (3, 6, 15). In addition, the risk of suicide increases when people do not work after retirement. Suicidal ideation was more prevalent among people with unstable or low financial status, low education level, and among those who live in low-income areas (9, 12, 15, 21). Financial difficulty during old age lowers QOL; this seems to be correlated with mental problems and, as a result, with suicidal ideation.

In terms of employment type, manual workers showed lower HRQOL and mental health than did non-manual workers. Significant differences were found in mobility and pain/discomfort, EQ-5D index and EQ-5D, and depression and suicidal ideation. This was supported by the findings that QOL was higher among the employed elderly (than the unemployed elderly) was permanent employees (than temporary employees), full-time employees (than part-time employees), and specialized professions (15). As physical laborers mostly work in small and poor environments and engage in simple and repetitive work, safety and healthcare issues are found; atypical work, which is the employment type of the vast majority of physical laborers, poses threats to mental health due to job insecurity (22). For both men and women, stressful physical labor was related to a higher suicide rate; highest suicide rates were found specifically in farming, fishing, and forestry, because these jobs easily make employees lonely and isolated, pay a low wage, and lead to low levels of mental well-being (23–26). Among the employed elderly, manual workers have severe problems related to mobility and pain/discomfort. Limited daily activities are closely related to elderly QOL (22, 27). Therefore, it is necessary to encourage safety education related to physical injury, diseases, and improvement of the physical environment for the elderly in order to regulate the level of physical labor and stress. In addition, health-related programs should be developed and introduced in order to maintain elderly workers’ mental and physical health.

In the analysis, factors that influenced HRQOL of people aged 65 yr and older included younger workers with higher educations who were non-smokers and non-drinkers. In addition, among the employed elderly, women and subjects completed high school, in comparison to those who completed only middle school, were likely to have a higher HRQOL. Men and smokers had a higher risk of depression, while a higher risk of suicidal ideation was found in poorly educated unemployed men who smoked. Among the employed elderly, smoking was related to a higher risk of suicidal ideation.

Occupation is an important link between education and income; education determines the type of occupation, which, in turn, determines the amount of financial compensation (28). This supports the finding that the elderly are more likely to have suicidal ideation when they have a low education and financial status levels (23, 29–31). Although among the elderly, suicidal ideation was more prevalent among women, the actual suicide rate was higher among men (31, 32), perhaps because of differences in gender roles, experience, and social participation (21, 33). For the male elderly, income, health, and life satisfaction influenced their QOL, while, for the female elderly, the presence of a spouse was more influential. This suggests the possibility that employment status may not have as significant an influence on QOL for the female elderly as it does for the male elderly (34). Different gender roles in society and gender-based inequality in the labor market may cause differences in employment in old age between men and women (33). In addition, differences in economic status, which influence suicidal ideation, between men and women can be explained by the argument that men are more likely to engage in economic activities than are women due to traditional responsibilities (35, 36). A low level of education influences socioeconomic status; men who must take financial responsibility for raising a family may experience more financial difficulty after retirement than women.

It is highly controversial whether smoking and/or drinking has an effect on suicidal ideation or attempts. While one study suggested that alcohol dependence was negatively correlated to suicidal ideation, another study argued that people with suicidal ideation due to depression have more suicidal ideation because of the alcohol (37, 38). Associations between smoking and suicide also have not been established; although smoking can lead to suicide because of deteriorated health and reduced serotonin, smokers can have depression or suicidal ideation after stopping smoking (39). However, the finding that suggests that the smoking elderly are more likely than the non-smoking elderly to have suicidal ideation is supported this study (21). Therefore, anti-smoking and anti-drinking education can be provided to improve HRQOL and mental health among the elderly. Various attempts, including developing mental health programs for the male elderly and employment policies, need to be made.

The results of this study suggest that participation of people aged 65 yr and older in productive activities through employment can be used as a tool to improve their HRQOL and mental health; elderly employment must be actively encouraged and supported by institutional arrangements. In addition, special management can be provided to the employee elderly involved in physical labor, as they are reported to have low HRQOL and high risk of suicidal ideation.

The major strengths of this study include the large size and representativeness of the sample used for examining the association of HRQOL and mental health by occupational status of people aged 65 yr and older and various instruments used for assessing HRQOL. Despite these strengths, this study had the following limitations. First, as a cross-sectional study, it cannot explain causal relationships, and, therefore, we propose a longitudinal study be conducted in the future. In addition, future studies should include a larger number of elderly subjects who are non-manual workers. Despite these limitations, we believe that this study provides meaningful findings, as it is the first study to examine the associations between HRQOL and mental health by occupational status among the elderly in South Korea.

## Conclusion

Job creation for the elderly is required in order to improve health-related QOL and prevent suicidal ideation in the elderly. Although even physical labor is necessary for the elderly to improve their QoL and mental health, considering their physical function and strength non-manual work or relatively easy work must be provided to the elderly.

## Ethical considerations

Ethical issues (Including plagiarism, informed consent, misconduct, data fabrication and/or falsification, double publication and/or submission, redundancy, etc.) have been completely observed by the authors.
